# The Effect of Smartphone-Based Application Learning on the Nursing Students’ Performance in Preventing Medication Errors in the Pediatric Units

**DOI:** 10.1097/pq9.0000000000000226

**Published:** 2019-11-28

**Authors:** Sima Pourteimour, Masumeh Hemmati MalsakPak, Madineh Jasemi, Samereh Eghtedar, Naser Parizad

**Affiliations:** From the * Patient Safety Research Center, Nursing and Midwifery School, Urmia University of Medical Sciences, Urmia, Iran; † Mother and Child Obesity Research Center, Nursing and Midwifery School, Urmia University of Medical Sciences, Urmia, Iran; ‡ Nursing and Midwifery School, Urmia University of Medical Sciences, Urmia, Iran

## Abstract

Medication errors (MEs) are potentially harmful patient safety events in all age groups. MEs are particularly hazardous in the pediatric population, especially with patients who require special attention due to the high incidence and severity of disease. This study aimed to determine the effect of a smartphone messenger application on nursing students’ learning regarding the prevention of MEs in pediatric patients.

**Methods:**

We performed this quasi-experimental study with 80 nursing students who were randomly divided into intervention and control groups. We collected the data using a researcher-made checklist. We conducted learning through Telegram, a smartphone messenger application (app), for 3 weeks. We analyzed data using SPSS version 16.0 by utilizing descriptive and inferential statistics, and *P* < 0.05 was considered to be significant.

**Results:**

The mean age of the students was 23.5 ± 2.9 years. The majority of mistakes related to MEs in the control groups included the lack of proper control of the following: high risk medication administration, medication incompatibility interactions, medication administration card, medication dose calculations, adverse drug event recognition, pharmaceutical name recognition during drug selection, aseptic and sterile technique adherence, microbore IV tubing flush practices, IV drip rate adjustment, and medication administration schedules. The mean scores of students’ performance were significantly different in the knowledge of preventing MEs between the 2 groups. (*P* = 0.022).

**Conclusions:**

Smartphone learning with the Telegram messenger app improves nursing student knowledge regarding the prevention of MEs in pediatric patients. We recommend that this form of learning be used in nursing schools to prevent errors related to medication ordering, dosing, and administration.

## INTRODUCTION

Medication errors (MEs) remain one of the most prevalent medical errors that lead to morbidity and mortality.^1^ MEs are used as an indicator for determining patient safety in hospitals.^2^ A ME is a preventable drug-related event due to any “failure in the treatment process that leads to the potential harm to the patients.”^3^ The majority of MEs reported by hospitals may be much lower than what can be tracked and accurately reported.^1^ In Iran, 8% of hospital treatments lead to complications, including adverse drug events, which are even higher than in the United States (2.4%–6.5%).^1,4^ In adults, reported incidences of MEs are between 1% and 30% of all hospital admissions, and 5% of these errors related to medication orders.^5^ The rate of MEs for hospitalized children maybe 3-fold higher than those for adults with significantly serious consequences.^6^ Such errors can be attributed to medication dose calculations based on the child’s weight, medication dilution, the challenge of children’s ability to communicate, high vulnerability of young and sick children, and secondary kidney and liver failure due to MEs. These errors may lead to increased hospital length of stay and associated costs.^5^

Ensuring the safe use of medications by adhering to the 5 rights of medication administration (right patient, right medication, right dose, right time, and right route) provides a framework for safe nursing practice.^7^ Recently, 5 additional items have been added, including right assessment, right to refuse, right evaluation, right education, and right administration.^8^ Medication administration is an important aspect of the patient’s care process and is a major component of nurses’ performance.^9^ Therefore, to reduce this preventable harm, children’s health systems and care providers should adopt and implement interventions to reduce pediatric MEs.^5^

Despite the efforts of university officials to educate safe medication administration, nursing students commit errors, and the research indicates that there is a high incidence of MEs in both nursing students and nurses.^10^ The goal of the nursing curriculum is to prepare students for their professional career as empowered and competent nurses who have the necessary knowledge, attitude, and skills to maintain and improve community health.^11^ However, sometimes, clinical environments are unpredictable, complex, and stressful for novice nurses. In turn, this fact can reduce their ability to think critically and function correctly.^12^ Thus, they may make the wrong decision regarding safe medication administration.^13^ So, nursing educators felt a strong need to find new and improved ways to train students.^14^ Meanwhile, nursing educators have accepted technology-based education as a solution.^13^ One of the technologies used most often is smartphone technology. The purpose of this technology in nursing education is to help educators enhance motivation and learning outcomes and to help nurses to improve their knowledge and skills.^15^

Until recently, few studies have been conducted to investigate the occurrence of MEs and the prevention of such errors in pediatric patients compared with the adult population.^16^ Nurse educators should determine more practical interventions that students can use to reduce MEs.^17^ Also, the impact of smartphone messenger applications (apps) like Telegram (a trademark of Telegram Messenger LLP) has not yet been investigated on nursing students’ performance in the prevention of MEs in pediatric patients in Iran. We conducted this study to determine the effect of smartphone messenger app learning techniques on the nursing students’ knowledge in the prevention of MEs in pediatric patients. In other words, we addressed the following question: “Does the use of a smartphone app like Telegram messenger have any impact on nursing students’ knowledge in the prevention of MEs in pediatric patients?”

## METHODS

### Participants and Study Setting

Participants were senior students enrolled in nursing school in Urmia, Iran. We assessed participants based on the following inclusion criteria: (1) students’ agreement to participate in the study, (2) students who complete institutional required courses, and (3) students with no prior learning experience about preventing MEs.

**Fig. 1.**
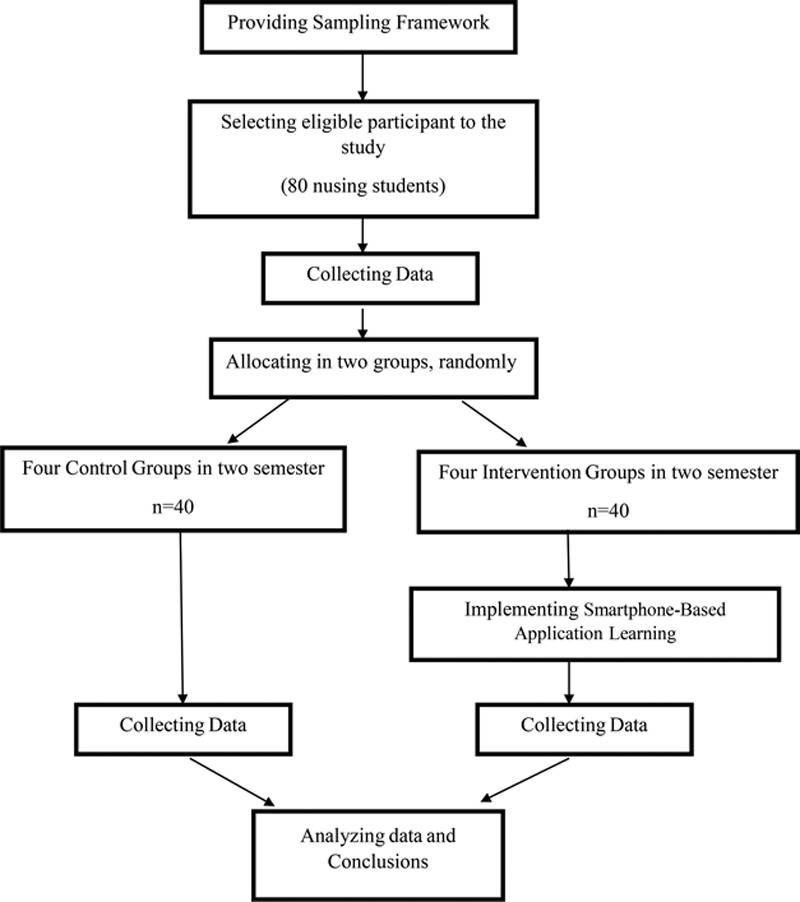
The flow diagram of the study based on Consort statement 2012. It indicates participants’ recruitment and intervention process.

We excluded nursing students who did not have a smartphone and failed to attend 2 sessions of group discussion during the intervention period. We used a random digit table to allocate 82 eligible participants to either a control or intervention group during 2 consecutive semesters. Two of the participants withdrew because they could not use the Telegram messenger app due to personal reasons. In total, we enrolled 80 participants in the study in 2 semesters. Thus, 40 participants divided into 4 groups, with 10 members each in every semester. We considered the first 2 groups of internship students as control groups and the last 2 groups as intervention groups in each semester to prevent data transmission between control and intervention groups. (Fig 1).

### Intervention

We conducted this quasi-experimental study from September 2015 to June 2016. We used the Telegram messenger app to educate nursing students. Telegram is a free social network in Iran. It is a messaging application that you can send any type of files such as text messages, picture and video free, fast, and secure.^18^ In Iran, most universities are making extensive use of new smartphone technology.^19^ Common smartphone messenger applications used for educational and learning purposes in Iran include Telegram(50%), WhatsApp (a trademark of Facebook, Inc.)(26%), Viber (a trademark of Rakuten) (7%), Instagram (a trademark of Facebook, Inc.) (2%), and others (12%).^20^ Telegram is the most popular messenger application in Iran, Uzbekistan, and Ethiopia. More than 40 million Iranians use Telegram app on their smartphones.^18^

The nursing students used this app to read and discuss the educational content provided by the researchers. We developed the educational content from a pediatric pharmacology textbook, the patient safety instruction book, and pediatric units practice guidelines in the prevention of MEs. A pediatrician and a pediatric nurse practitioner approved the educational contents before starting the intervention. These didactic materials were delivered to the participant through a Telegram messenger in 9 sessions with 60 minutes each. Electronic education was conducted 3 times a week (Saturday, Monday, and Wednesday) in a Telegram channel created for online small group interactions and exchange of ideas. For more information regarding educational contents, please see Table 1.

**Table 1.**
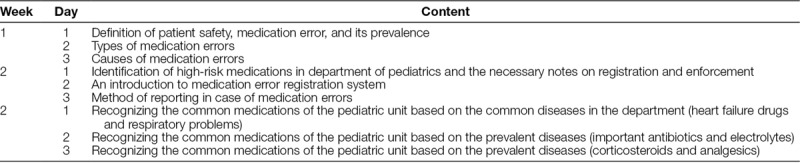
Learning Content Based on Training Sessions Through Telegram Messenger Application

To review the contents of the previous session, we conducted a question and answer review in the first 15 minutes of each session. We sent the content of the upcoming session to nursing students in PDF format to a Telegram channel at the end of each educational session. Then, we asked students to refer to the channel and study the new contents. In the control group, students received only routine training by their instructors.

### Measurements/Instruments

Data collection tools consisted of 2 parts: a demographic information form and the performance checklist, which we prepared based on the patient safety protocol and safe medication administration guidelines in pediatric patients. It included 50 items with a 2-point scale (observed = 0, not observed = 1). Five nursing professors and 2 pharmacists confirmed the face validity of the checklist. We used Laws Table to confirm the numeric value of the content validity ratio. We kept the items whose ratio was higher than 0.42. The content validity index was 0.80 based on Waltz and Bausell.^21^ We measured the correlation coefficient (*r* = 0.89) to check the interrater reliability of the checklist after 10 nursing students filled out the checklist.

We considered age, gender, pharmacology score, grade point average, MEs, participants’ experience of observing MEs, and their participation in a course about patient safety as demographic information.

### Data Collection and Procedures

We recruited students who wished to participate in the study. On the first day of the internship program, participants arrived at the pediatric unit at their scheduled time. Participants received an orientation session before beginning the study. We conducted performance assessments one-on-one and allocated 20 minutes for each student during the first week of internships. The participants in intervention groups used their smartphone messenger app repeatedly for 3 weeks. Also, each participant was only able to log into his/her smartphone content using the given personal phone number. The researchers (a nursing professor and a registered nurse with >5 years of clinical experience) administered the pretest and post-test.

### Ethical Considerations

The Ethics Committee of Urmia University of Medical Sciences approved this study (Approval code: Ir.umsu.rec. 242. 1394). During participant recruitment, the primary researcher who never met the students conducted informed consent, to prevent any possible coercive pressure.

### Data Analysis

All statistical analyses were performed using SPSS, version 16.0 (IBM Corp., Somers, N.Y.). Descriptive (mean, SD) and inferential (independent *t* test, chi-square tests, and Fisher’s exact test) statistics, were used to analyze data. We used the Shapiro–Wilk test to identify the homogeneity of participants’ characteristics. Statistical significance was set at *P* < 0.05.

## RESULTS

The research groups were not significantly different in terms of age, gender, pharmacology scores, history of MEs in the control, and intervention groups (Table 2).

**Table 2.**
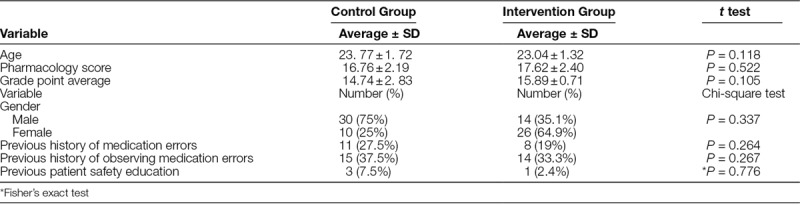
Comparison of Demographic Characteristics Between 2 Intervention and Control Groups

The most common mistake made by the nursing students regarding the prevention of MEs in the control group included:

1) The failure to control medication incompatibility interactions with other medications and child’s diet;
2) The lack of control over high-risk medications and during the administration of these medications;
3) The lack of proper control of the medication administration card;
4) The failure to calculate the medication dose accurately during the preparation;
5) The inappropriate IV drip rate adjustment and medication administration schedules.

Some of the most common nursing student mistakes in the intervention group were the lack of proper control of the medication administration card, the failure to calculate the medication dose accurately during the preparation, and the failure to inform the doctor, responsible nurse, or instructor in the cases of observing the complications and symptoms of medication overdoses in the patient. A higher frequency of errors occurred in the control groups suggesting that e-learning with Telegram messenger app had a significant effect on the students’ performance in the intervention groups (Table 3). There was a significant difference between the mean scores of students’ performance in preventing MEs in the control (34.22 ± 5.99) and intervention (46.66 ± 6.06) groups after the smartphone messenger app learning (*P* = 0.022).

**Table 3.**
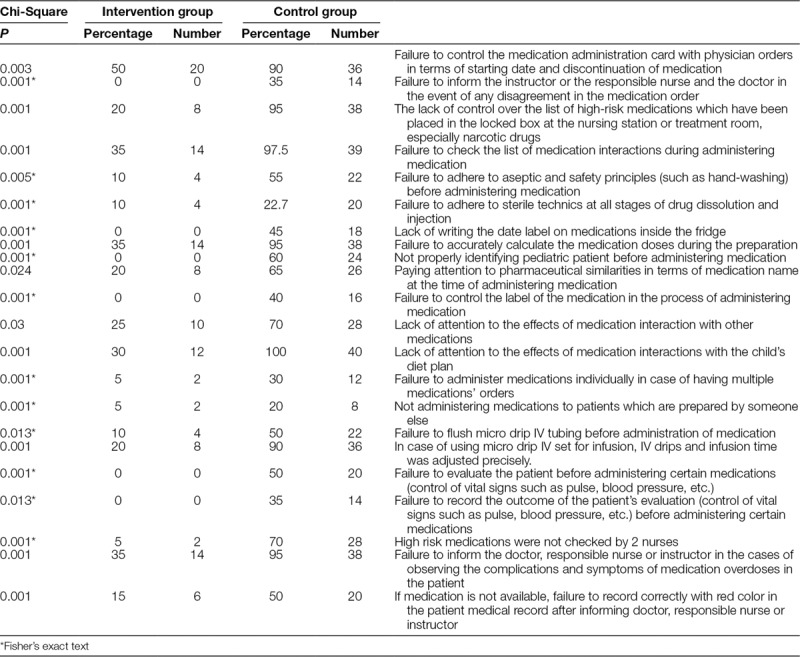
Comparison of the Highest Frequency of MEs of Students in the Pediatric Units

## DISCUSSION

The results of this study show that the smartphone messenger app has a positive effect on the knowledge and performance of nursing students that may result in the prevention of MEs. In line with another recent study, the poor performance of nursing students in preventing MEs resulted from lack of pharmacological information related to medication interactions with other medication and the child’s diet and lack of knowledge regarding high-risk medications.^22^ Baghaei et al^23^ reported that the most important MEs among nursing students is related to the inappropriate administration of intravenous medications, especially antibiotics. They believed that the most significant reason for such errors is insufficient knowledge of students in pharmacology.^23^ Similar to this finding, a recent study showed short staffing and poor pharmacology knowledge of nurses are among the factors that lead to MEs in neonatal units.^24^

This study also showed that the most common errors in the pediatric units were related to the lack of proper control of the medication administration cards as well as miscalculating the medication doses. Azarabad et al^25^ reported a similar result among the students’ MEs in the operating room. A recent study reported that MEs related to pediatric dosing error was 23%, similar to our findings. In this regard, Park and Kim^26^ indicated that using the smartphone app was effective in improving nursing students’ self-efficacy for drug dosage calculation. It is possible to prevent the occurrence of MEs and medication overdose secondary to such errors with the appropriate strategy, such as smartphone app training of medication dosage calculation.

In this study, most students did not pay attention to the common symptoms of medication side effects in the pediatric unit. Similar to our findings, in every 1,000 pediatric patients in Japan, 37.8% faced adverse drug events due to MEs (65.1%). More than half of these patients had serious complications which monitoring medication use processes could be the most significant factor in improving medication safety.^27^ As noted above, reviewing and controlling drug antidotes, and the complications and symptoms of medication overdoses should be considered as an important element in reducing MEs in pediatric units.

In our study, the majority of nursing students had difficulty adjusting IV drips and calculating infusion time. In a study conducted by Vaismoradi et al, nursing students voiced that only the theoretical content related to medications (such as medications categories, indication, side effects, etc.) are discussed in pharmacology classes. There is no mention of the practical aspects of medication management.^28^ Consistent with this study is a recent study that showed the performance of nearly half of the nurses was inappropriate for infusion of IV medications, and 24% of the nurses administered medications to patients at the wrong time.^29^ A recent study conducted in Isfahan hospitals in Iran confirmed our findings.^30^ Using this smartphone app reduced MEs, medication preparation, and delivery time during resuscitation in the pediatric unit.^31^ Thus, it seems that there is the potential to improve pediatric clinical practice by utilizing modern technology in training of MEs prevention as a medication administration standard for students and nurses.

In the current study, nursing students did not pay attention to similarities in pharmaceutical names during the preparation of medication. Some studies have highlighted this issue in the guidelines for the prevention of MEs.^32,33^ Therefore, the effect of pharmaceutical names similarities in the occurrence of MEs is undeniable, and checking the name of prescribed medications should be emphasized before administration.

Our study suggests that the lack of knowledge regarding the need to properly flush micro drip IV tubing before medication administration and adhere to aseptic and sterile techniques may contribute to common errors in pediatric patients. Although it is essential to observe all of the above before administering the medication, sometimes nursing students commit MEs due to heavy workload and a crowded unit. A recent study confirms these possibilities.^22^

The results also indicated that MEs might occur while administering medication prepared by others. Consistent with our finding, recent studies showed that sometimes patients and their parents were asked to administer their medications.^34,35^ That practice is unsafe and could lead to MEs. Hence, nursing student knowledge regarding the proper preparation and administration of medication may improve medication safety. We should include and emphasize this information in the MEs prevention protocol.

## LIMITATIONS

One of the limitations of this study is the availability of other educational resources to nursing students. To overcome this limitation, nursing students shared their questions with the researchers and other students with a smartphone messenger app or internet (simultaneous conversation in the Telegram chat room or sending an email). On the other hand, the researchers tried to provide content from the latest and the most reliable sources to cover all the educational needs of students in preventing MEs in a way that they do not need to refer to other educational resources. Also, there is the possibility of contact and transferring information between the 2 groups during the study. Thus, the researchers considered the first and second groups as the control groups and third and fourth groups as the intervention groups to prevent this data transfer. Based on the findings of this study, the researchers suggest further studies by utilizing other educational content such as film, sound, and animation as well as combining different learning methods such as e-learning, the lectures, etc.

## CONCLUSIONS

Pediatric patients are at risk for MEs. This study brings attention to the effectiveness and significance of the integration of mobile technologies such as smartphone apps into teaching, learning, and clinical practice of nursing students. Second, it may help students to reduce the occurrence of MEs, and improve drug preparation time and delivery time. Utilizing innovative smartphone apps is highly recommended, especially in pediatric units. In general, this study shows that nursing students’ knowledge of ME improved by utilizing smartphone messenger app.

## ACKNOWLEDGMENT

This article is part of a project in Urmia nursing school. The authors would like to thank the research deputy of Urmia University of Medical Science for financially supporting the project. They would also express their profound gratitude toward the nursing students participating in the research, as well as, Mariam Angelica Parizad for her review of the article and writing assistance.
